# Supplementary material for: Snapshot prey spectrum analysis of the phylogenetically early-diverging carnivorous Utricularia multifida from U. section Polypompholyx (Lentibulariaceae)
Source: PLoS One. 2021 Apr 7;16(4):e0249976. doi: 10.1371/journal.pone.0249976 (PMC8026053; doi:10.1371/journal.pone.0249976)
Supplement: S1 Table — (PDF) [file pone.0249976.s001.pdf]

SUPPORTING INFORMATION FOR

Snapshot prey spectrum analysis of the phylogenetically early-diverging carnivorous *Utricularia multifida* from *U*. section *Polypompholyx* (Lentibulariaceae)

Martin Horstmann[1], Andreas Fleischmann[2], Ralph Tollrian[1], Simon Poppinga[3,4]*

[1] Department of Animal Ecology, Evolution and Biodiversity, Ruhr-University Bochum, D-44780 Bochum, Germany

[2] Botanische Staatssammlung München, D-80638 München, Germany

[3] Plant Biomechanics Group and Botanic Garden, University of Freiburg, D-79104 Freiburg im Breisgau, Germany

[4] Freiburg Materials Research Center, University of Freiburg, D-79104 Freiburg im Breisgau, Germany

** Corresponding author*

Email: simon.poppinga@biologie.uni-freiburg.de (SP)

SI Table: *Utricularia multifida* trap length and width measurements.

Plant	Trap	Length [µm]	Width [µm]
1	1	2549	2292
1	2	2026	1831
1	3	2445	2042
1	4	2273	2082
1	5	2387	1888
2	1	2201	2003
2	2	2267	2042
2	3	1783	1542
2	4	2022	1661
2	5	2733	1687
